# Antioxidant and phytochemical activities of *Amaranthus caudatus* L. harvested from different soils at various growth stages

**DOI:** 10.1038/s41598-019-49276-w

**Published:** 2019-09-10

**Authors:** Muhali Olaide Jimoh, Anthony Jide Afolayan, Francis Bayo Lewu

**Affiliations:** 1 Medicinal Plants and Economic Development (MPED) Research Centre, Department of Botany, University of Fort Hare, Alice, 5700 South Africa; 2 Department of Agriculture, Cape Peninsula University of Technology, Wellington Campus, Wellington, 7654 Cape Town South Africa

**Keywords:** Ecology, Plant sciences

## Abstract

This study aimed at profiling the biological activities of *Amaranthus caudatus* cultivated on different soils in a glasshouse experiment. Five soil types namely; sandy clay loam, silty clay loam, clayey loam, loam and control (unfractionated soil) were experimentally formulated from primary particles of clay, sand and silt following the United State Department of Agriculture’s (USDA) soil triangle technique. After harvesting at pre-flowering (61 days after planting), flowering (71 days after planting) and post-flowering (91 days after planting) stages, crude extracts were obtained with water and ethanol. Total flavonoids, phenolic and proanthocyanidin contents of the extracts, as well as their biological activities, were determined using 2,2′-azino-bis(3-ethylbenzothiazoline-6-sulphonic acid) (ABTS), 2,2 diphenyl-1-picrylhydrazyl ethanol (DPPH), nitric oxide and phosphomolybdate assays. It was observed that biological activity of *A. caudatus* varied with soil types, stages of maturity and solvents of extraction. The highest phytochemical yield was recorded in ethanolic extracts of clayey loam harvested prior to flowering and the same trend was replicated in the antioxidant properties of the plant. For optimal biological activity, it is recommended that clayey loam soil should be used for cultivation of *A. caudatus* and harvest should be made near flowering to capture high phytochemical yield from the species.

## Introduction

Natural compounds such as storage lipids, fragrances, essential oils, flavonoids, polyphenols, and pharmaceutics extracted from plants, have been extensively investigated for their food value and are used as precursors by cosmetics and pharmaceutical industries^1,2^. Over a hundred thousand of these secondary metabolites are either biosynthesised via acetate pathway or derived from alkaloids, phenylpropanoids and isoprenoids^3,4^. The biological activity of these natural products is mostly driven by the evolutionary process as related plant families usually make use of analogous chemical structures in building up resistance against diseases to improve their defence mechanism^5,6^.

A combination of soil and other environmental factors are capable of altering plant structure and its phytochemical activity, thereby affecting the interaction between the plant and the environment and ultimately, the depot of bioactive compounds in the leaves^7,8^. In addition, the variation observed in the germination response of *A. caudatus* to soil types is an indication that soil type, texture, water relation and mineral composition of the soil affect growth performance of the plant and most likely, the number of secondary metabolites produced by the plant^8^. Likewise, accumulation of vitamins and phenolic compounds has been reported to have a direct relationship with soil mineral content and it relates in inverse order with the growth rate of plants^9^.

As part of normal cellular activity, production of free radicals results from both endogenous and exogenous reactions taking place in a biological system. Most of these free radicals are short-lived derivatives of oxygen and they possess unpaired electrons capable of existing independent of other molecules^10–12^. *A. caudatus* is an ancient crop with protein content greater than most cereals^13^. It has been widely reported in the literature as an important source of bioactive compounds such as lectin, phenolics and flavonoids capable of trapping free electrons^14–16^.

The potentials of crude extract of *A*. *caudatus* in trapping free radicals associated with oxidative damages have been deliberated extensively^15,17–19^; however, there is a dearth of information on the effect of formulated soils and stages of harvest on its phytochemical and antioxidant properties. This research, therefore aimed at profiling phytochemical and antioxidant activities of *A. caudatus* cultivated on different soils in relation to stages of harvest. Further, variation in the quantity of phytochemicals and antioxidant components of the plant was investigated at various stages of harvest with a view to understanding the soil with the highest yield of bioactive compounds and at a particular growth stage.

## Materials and Methods

### Experimental soil formulation

A heap of topsoil collected from the University of Fort Hare’s Research Farm was air-dried under shade for four weeks. The dried soil was later filtered with iron sieves of the designated mesh into parent particle sizes of clay (<2 µm), silt (<50–2 µm) and sand (<2000–50 µm). Four experimental soil types were formulated by mixing sieved soil particles in relative proportions recommended by the USDA’s soil texture triangle (Table 1) and used for cultivation alongside with the control soil.

**Table 1:** Experimental soil formulation in proportions proposed by USDA soil texture triangle technique^52^.

S/N	Soil types	% Sand	% Silt	% Clay
1	Control soil (SF_1_)	unfractionated	unfractionated	unfractionated
2	Sandy Clay Loam (SF_2_)	66	13	21
3	Silty Clay Loam (SF_3_)	10	60	30
4	Clayey Loam (SF_4_)	36	30	34
5	Loam (SF_5_)	40	40	20

### Plant material collection and processing

Viable seeds of *A. caudatus* were cultivated in summer (between October 2017 and January 2018). At first, seeds were propagated in five seed trays, each filled near brim with different soils. Each seed tray measuring 65 × 100 cm^2^ has 200 cells embedded in it. A seed each was planted in each cell and growth was monitored for four weeks. At the fourth week, seedlings were transplanted to the respective soil types (about 7 kg) already heaped in pots filled to the near brim. A total number of 240 pots were used and the pot experiment was conducted and organized in three replicates in a Completely Randomised Design (CRD) inside the controlled glasshouse of Botany Department, University of Fort Hare, South Africa. Plants were irrigated twice daily (morning and evening). Shoots of *A. caudatus* (leaf and herbaceous stem only) were harvested at three growth stages namely; pre-flowering, flowering and post-flowering. The plant samples were sorted according to soil types and stages of harvest and dried in an oven set at 40 °C until a constant weight was attained. After drying, samples were pulverized and 200 g each of the pulverized samples was kept in an airtight container at 4 °C in a refrigerator for further treatment.

### Extraction procedure

Distilled water and ethanol were used for extraction. The solvents were chosen based on the food value of aqueous extract and wide report on medicinal effects of ethanolic extract. 60 g each of the powdered samples were soaked in 1000 mL of different solvents in a conical flask and shaken at 120 rpm for 48 hours in a mechanical shaker (Orbital Incubator Shaker, Gallenkamp). The crude extract was filtered through a Whatman No. 1 filter paper placed in a Buchner funnel connected to a vacuum pump. The resulting ethanolic filtrate was concentrated to dryness using a rotary evaporator (Strike-202 Steroglass, Italy) set at 78 °C. Aqueous filtrate collected was chilled at −40 °C in a refrigerant (PolyScience AD15R-40-A12E, USA) and concentrated to dryness within 24 hours using a freeze dryer (Savant vapour trap, RV-T41404, USA).

### Chemicals and reagents

All chemicals used in this study were of analytical grades and were procured from Merck Millipore and Sigma-Aldrich, Johannesburg, South Africa. These include; ABTS (2,2′-azino-bis(3-ethylbenzothiazoline-6-sulphonic acid), acetone, aluminum trichloride (AlCl_3_), ammonia solution, ammonium molybdate, anhydrous sodium carbonate (Na_2_CO_3_), ascorbic acid, butylated hydroxyl toluene (BHT), diethyl ether, DPPH- 2,2 diphenyl-1-picrylhydrazyl ethanol, ferric chloride (FeCl_2_), Folin-Ciocalteu, Gallic acid, glacial acetic acid (CH_3_ COOH), hydrochloric acid, methanol, n-butanol, phosphate buffer, potassium acetate (CH_3_ CO_2_K), potassium ferricyanide (K_3_Fe(CN)_6_), rutin, sodium chloride, sodium hydroxide, sodium nitroprusside (Na_2_[Fe(CN)_5_ NO]_2_H_2_O), sodium nitrite (NaNO_2_), sodium phosphate, trichloroacetic acid (TCA), and vanillin.

### Readings and statistical analysis

All absorbance was read at specified wavelengths using the USA made Diagnostic Automation, Inc microplate reader (SN: 259557). IC_50_ (mg/mL), the concentration of water and ethanol extracts at which 50% inhibition or scavenging occurred was estimated at 95% confidence interval using MINITAB 17 statistical package. The variance of various mean phytochemical values of ethanol and aqueous extracts from different soils were also computed with MINITAB 17 and means were separated with Fischer’s Least Significant Difference (LSD) test at α = 0.05.

### Phytochemical profiling

#### Total flavonoids

The total flavonoid content was measured following the aluminium chloride spectrophotometric assay described by^20^ with slight modification. The assay is based on the quantification of the yellow-orange colour of flavonoid-AlCl_3_ complex arising from the reaction between flavonoids and AlCl_3._ A stock of the plant extracts and standard (quercetin) was prepared in 1 mg/mL of the solvent of dissolution. Graded concentrations of quercetin (0.2–1.0 mg/mL) were prepared and 0.5 mL of each concentration (quercetin) and the extract was pipetted into separate test tubes. Afterwards, 2 mL of distilled water was added to the test tubes followed by 0.15 mL of 5% NaNO_2_. The mixture was vortexed and allowed to stand for 6 min. After the waiting period, 0.15 mL of 10% AlCl_3_ was added to the mixture together with 1 mL of 1 M NaOH which was added 5 min later. The solution was made up to 5 mL with the addition of distilled water and incubated at 40 °C for 20 min. After incubation, 300 µL each of extracts and graded concentrations of quercetin was pipetted in triplicates into designated wells of the microplate. The absorbance of the pipetted mixture was recorded at 430 nm with a microplate reader. Total flavonoid content was estimated as mg/g of quercetin equivalent (QE/g) from the standard calibration curve *y* = *0.3658x* + *0.0356, R*^2^ = *0.9618* using the equation [$\frac{CV}{M}$]. C is the concentration extrapolated from the standard linear graph, V is the volume of extract in mL and M is the mass of the extract used expressed in gram (g).

#### Total phenolic content

The total phenolic content of the extract was determined using Folin-Ciocalteu’s procedure adopted by^21^ with some modifications. The standard used was gallic acid, prepared as 1 mg/mL in methanol. One mg of the crude extract was dissolved in 1 mL of methanol and the standard’s concentration was graded in a series of 0.2–1.0 mg/mL. To 0.5 mL of extract and standards in separate test tubes, 2.5 mL of Folin-Ciocalteu reagent was added. Thereafter, 2 mL of anhydrous Na_2_CO_3_ (7.5% w/v) was added. The entire mixture was vortexed and incubated at 40 °C for 30 min. Immediately after incubation, 300 µL each of the extract solutions and graded concentrations of gallic acid was pipetted in triplicates into a 96-welled microplate and absorbance was measured at 750 nm with the aid of a microplate reader made in the USA by Diagnostic Automation, Inc (SN: 259557). The total phenolic content was evaluated as mg of gallic acid equivalent (GAE) per gram of crude extract deduced from the standard linear graph *y* = *0*.22*81x* − *0.0264, R*^*2*^ = *0.964* using the equation $\frac{CV}{M}$ given above.

#### Proanthocyanidin content (condensed tannin)

The total proanthocyanidin content was evaluated as described in^2^ with minor modifications. 0.5 mL each of the stock (extract and standard) was reacted with 3 mL of vanillin (4% w/v), then with 1.5 mL of HCl. The resulting solution was vortexed and incubated at room temperature for 15 min. 300 µL each of the extract and graded concentrations of catechin was dispensed in microplate in three replicates and absorbance was read at 500 nm with the aid of a microplate reader. The total proanthocyanidin content was estimated as mg of catechin equivalent (CE)/g of the crude extract obtained from the calibration curve *y* = *4.7541x* − *0.4801, R*^*2*^ = *0.9437* from the formula $\frac{CV}{M}$ used above.

#### Evaluation of antioxidant activity

The antioxidant activities of *A. caudatus* were estimated using nitric oxide (NO) inhibitory activity, DPPH free radical scavenging, phosphomolybdate - total antioxidant capacity (TAC) and ABTS free radical scavenging assays. Estimations were made against standard antioxidant compounds such as rutin and BHT.

#### Inhibition of nitric oxide (NO) production

The percentage inhibition of plant extracts against NO radicals was determined from the protocol described by Unuofin *et al*.^22^. A volume of 10 mM sodium nitroprusside prepared in 0.5 mM phosphate buffer saline (pH 7.4) was added to crude extracts of the plant and standard compounds (rutin and BHT) at different concentrations (0.025–0.400 mg/mL). The mixture was incubated at 27 °C for 2.5 hrs. After incubation, Griess reagent containing 0.33% sulphanilamide dissolved in 20% glacial acetic acid and mixed with 0.1% w/v of 1-naphthylethylenediamine in ratio 1:1 was added to the solution and allowed to react at room temperature for 30 min. A 300 µL volume each of graded concentrations of extracts and standard was dispensed in 96- welled microplate in three replicates and absorbance was read at 540 nm. The equation below was used to estimate the percentage NO inhibition of the extract and standard.

1 $$ \% \,{\rm{NO}}\,{\rm{inhibition}}=[\frac{{\rm{Abs}}\,{\rm{sample}}-{\rm{Abs}}\,{\rm{control}}}{{\rm{Abs}}\,{\rm{sample}}}]\,\ast \,100$$

where;

Abs_sample_ = absorbance of NO radical + extract or standard.

Abs_control_ = absorbance of NO radical + methanol.

#### DPPH free radical scavenging activity

The free radical scavenging capacity of test samples was estimated following^23,24^ with slight modifications. The percentage of DPPH radicals scavenged by the samples was calculated using Prieto’s DPPH microplate assay^25^. A solution of 0.135 mM of 2, 2-diphenyl-1-picrylhydrazyl was prepared in a dark bottle using methanol. This was mixed with serially diluted concentrations of the test sample and rutin standard in equivalence of 0.08, 0.04, 0.02, 0.01, 0.005 mg/mL respectively in ratio 1:1. The mixture was vortexed and incubated at room temperature for 30 mins. Thereafter, a volume of 300 µL dispensed in a 96-welled microplate was read at an absorbance of 517 nM. The scavenging activity of the tested samples was extrapolated in the inhibitory percentage of DPPH using the equation;

2 $$ \% \,{\rm{DPPH}}\,{\rm{scavenging}}=[\,\frac{({\rm{Abs}}\,{\rm{sample}}+{\rm{DPPH}})-({\rm{Abs}}\,{\rm{sample}}\,{\rm{blank}})}{({\rm{Abs}}\,{\rm{DPPH}})-({\rm{Abs}}\,{\rm{solvent}})]}]\ast 100\,$$

The half-inhibitory concentration (IC_50_) of the extracts was computed from the graph of mean percentage DPPH inhibitory activity (taken in triplicates) against the equivalent of tested samples concentrations in linear regression.

#### Phosphomolybdenum inhibition or total antioxidant capacity (TAC) assay

The total antioxidant capacity of the plant fractions was assayed following the method described in^26^. A volume of 0.3 mL of different solvent grades of crude extracts was added to 3 mL of reagent solution (4 mM ammonium molybdate, 0.6 M sulphuric acid, and 28 mM sodium phosphate) in glass test tubes. The stocked tubes were incubated in a water bath set at 95 °C for 90 min. After incubation, the mixture was allowed to cool off to room temperature and absorbance of the 300 µL of the mixture dispensed in a microplate was measured on a microplate reader at 695 nm. Gallic acid and rutin were used as standard and high percentage inhibition indicates high antioxidant activity and vice versa. The percentage inhibition was thus calculated as;

3 $$ \% \,{\rm{TAC}}\,{\rm{inhibition}}=[\frac{{\rm{Abs}}\,{\rm{sample}}-{\rm{Abs}}\,{\rm{control}}}{{\rm{Abs}}\,{\rm{sample}}}]\,\ast \,100\,$$

where;

Abs_sample_ is the absorbance of sample + reagent solution.

Abs_control_ is the absorbance of reagent solution and methanol.

#### ABTS free radical scavenging capacity

This assay was carried out following the protocol adopted in^23^ with modifications. In this method, the ABTS radical was generated by reacting equal volume of 2.45 mM of K_2_S_2_O_8_ and 7 mM ABTS solution prepared in methanol. The resulting solution was kept in the dark for 12–18hrs at room temperature and further diluted with methanol in a ratio 1:50 until an absorbance of 0.700 ± 0.003 was attained at 734 nm. Equal volumes of plant extracts and standard drugs of different concentrations were reacted with ABTS^**+**^ (1:1 v/v), left in the dark for 6 min and absorbance was taken at 734 nm. The percentage ABTS^**+**^ scavenging capacity of the crude extracts and standard (BHT and rutin) was calculated using the formula;

4 $$ \% \,{\rm{ABTS}}\,{\rm{scavenging}}=100-[\frac{{\rm{Abs}}\,{\rm{sample}}-{\rm{Abs}}\,{\rm{sample}}\,{\rm{blank}}}{{\rm{Abs}}\,{\rm{control}}}]\,\times 100$$

#### Statistical analysis

All readings were taken in triplicates and analysed with MINITAB 17 statistical software. The mean values of tested samples were compared using a one-way analysis of variance (ANOVA). At p < 0.05, means were considered significantly different and ranked using Fisher’s Least Significant Difference (LSD) paired wise comparison assuming equal means. Data were expressed as Means ± Standard Deviation.

## Results

### Effects of soil types on phytochemical content

#### Total phenolic content

**Figure 1:**
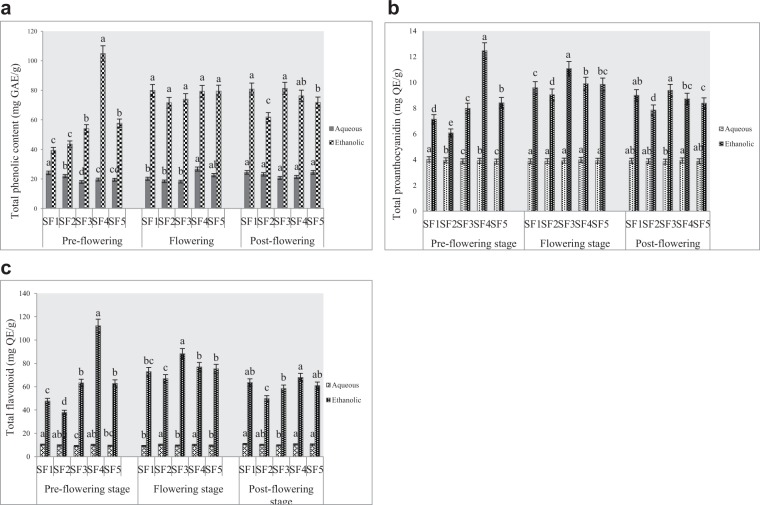
(**a)** Effects of soil types on total phenolic content of *A. caudatus* at various stages of harvest. SF_1_ = control soil, SF_2_ = sandy clayey loam, SF_3_ = silty clayey loam, SF_4_ = clayey loam, SF_5_ = loam. (**b**) Effects of soil types on condensed tannin in *A. caudatus* at various stages of harvest. SF_1_ = control soil, SF_2_ = sandy clayey loam, SF_3_ = silty clayey loam, SF_4_ = clayey loam, SF_5_ = loam. (**c)** Effects of soil types on flavonoids in A. caudatus at various stages of harvest. SF1 = control soil, SF2 = sandy clayey loam, SF3 = silty clayey loam, SF4 = clayey loam, SF5 = loam.

The total phenolic content was expressed as mg/g of the test samples in gallic acid equivalent (GAE) as presented in Fig. 1a below. In all extracts, phenolic contents were higher in ethanolic than aqueous samples. At the pre-flowering stage, ethanolic extracts of harvest from clayey loam (SF_4_) had the highest phenolic content. Also, phenolic content was highest in the aqueous samples of control soil. From the result obtained, there is no significant difference (p < 0.05) in the phenolic content of ethanolic extracts at the flowering stage but an aqueous extract of the clayey loam produced the highest phenol while silty clayey loam yielded the lowest at flowering stage. At post-flowering, there were no significant differences in phenolic compositions of aqueous extracts derived from all soils. While control and silty clay loam yielded highest of phenols in ethanolic extracts, least amount was obtained in the sandy clay loam. This assay shows that the most active sample was ethanol extract from clayey loam at the pre-flowering stage.

#### Total proanthocyanidin (condensed tannin)

This was estimated as mg/g of crude extracts in catechin equivalent (CE) represented in Fig. 1b below. The results also revealed that ethanolic extracts yielded more condensed tannin than aqueous extracts in all soil types and at all stages of maturity under study. At the pre-flowering stage, clayey loam yielded the highest proanthocyanidin than other soils while the least content was recorded in sandy clayey loam. At p < 0.05, aqueous extracts showed no significant difference in proanthocyanidin content at flowering stage whereas ethanolic extracts from silty clayey loam had the highest yield of proanthocyanidin, while lowest yield was obtained from sandy clayey loam. At post flowering, the trends obtained in proanthocyanidin content of ethanolic samples were replicated although, at this stage, the phytochemical was highest significantly in aqueous samples of clayey loam. This assay also indicates that the most active sample was ethanol extract from clayey loam at the pre-flowering stage.

#### Total flavonoids

This was expressed as mg QE/g of tested samples as shown in Fig. 1c below. As obtained in phenol and condensed tannins, ethanolic extracts yielded more flavonoids than aqueous samples. At the pre-flowering stage, ethanolic samples of clayey loam soil yielded the highest flavonoids and the lowest value was recorded in sandy clayey loam soil. At the flowering stage, the ethanolic extract had the highest flavonoid on silty clayey loam and the least amount was recorded in sandy clayey loam. In the aqueous samples, flavonoid values ranged from 9.26 ± 0.31 to 10.25 ± 0.13 (mg QE/g) in the control and sandy clayey loam soils respectively. The lowest amount of flavonoid was quantified at the post-flowering stage, however, ethanolic extract from clayey loam produced the highest flavonoids whereas, in aqueous extracts, there were no significant differences in control, clayey loam and loamy soils samples.

### Antioxidant activities

#### Inhibition of nitric oxide production

**Figure 2:**
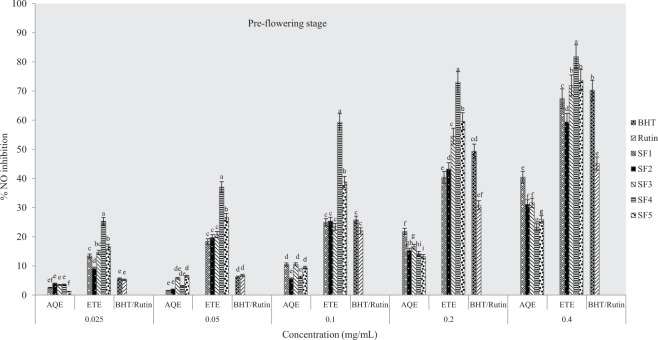
Effects of soil types on % inhibition of NO in *A. caudatus* at the pre-flowering stage. AQE = aqueous extract, ETE = ethanolic extract, BHT and rutin were standard used.

At all growth stages studied, the highest percentage of NO inhibitory capacity of *A. caudatus* extracts was observed in ethanolic extracts of clayey loam samples for all concentrations. Standard drugs used were BHT and rutin and higher inhibitions were found in BHT except at 0.05 mg/mL concentration. Results obtained further show that the inhibitory activities of the plant extracts were solvent and concentration-dependent. For the pre-flowering stage (Fig. 2), the highest inhibition was recorded in ethanolic samples of clayey loam soil. It was also observed that clayey loam soil extract inhibits NO more than both standard drugs (BHT and rutin) used. On the other hand, the aqueous extracts showed the highest inhibition in control soil which is comparable to the activity of rutin.

**Figure 3:**
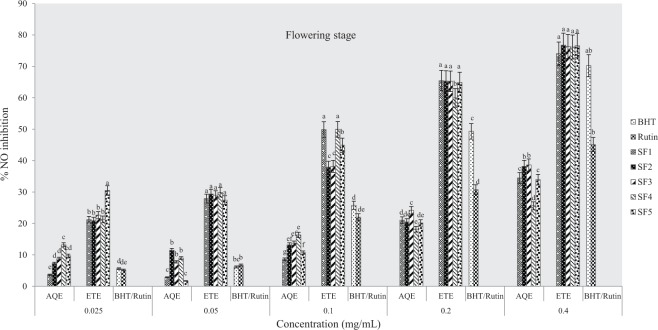
Effects of soil types on % inhibition of NO in *A. caudatus* at flowering stage. AQE = aqueous extract, ETE = ethanolic extract, BHT and rutin were standard used.

**Figure 4:**
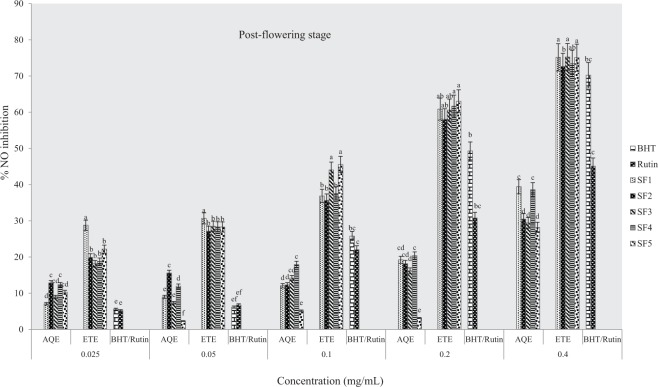
Effects of soil types on % inhibition of NO in *A. caudatus* at the post-flowering stage. AQE = aqueous extract, ETE = ethanolic extract, BHT and rutin were standard used.

At flowering stage (Fig. 3), results showed that ethanolic extracts inhibited NO radicals more than the standards (BHT and rutin) and aqueous samples although at highest concentrations used, there was no significance in the degree of inhibition between samples from different soils (P < 0.05). As observed in other growth stages, the inhibitory activities were more potent in the ethanolic extracts at post-flowering stage compared to standard drugs and water samples at various concentrations used (Fig. 4).

The IC_50_ values for ethanolic extracts ranged as follow; SF_4_ > SF_5_ > SF_3_ > SF_1_ > SF_2_ at pre-flowering stage, SF_4_ > SF_1_ > SF_5_ > SF_3_ > SF_2_ at flowering stage and SF_5_ > SF_3_ > SF_4_ = SF_1_ > SF_2_ at post flowering growth stages while for aqueous extracts (Figs 2–4), IC_50_ values were in the order SF_1_ > SF_2_ > SF_3_ > SF_5_ > SF_4_, SF_3_ > SF_2_ > SF_5_ > SF_5_ > SF_4_ and SF_5_ > SF_1_ > SF_4_ > SF_3_ > SF_2_ respectively at pre-flowering, flowering and post-flowering stages (Table 2). This assay indicates that the most active extract was from clayey loam (SF_4_) at the pre-flowering stage. In addition, results showed that the extract was more potent than standard drugs (BHT and rutin).

**Table 2:** IC_50_ values for % NO inhibitory activity of *A. caudatus* cultivated on formulated soils.

Soil types	Pre-flowering				Flowering				Post-flowering			
	IC_50 aq-1_	R_2_	IC_50 eth-1_	R^2^	IC_50 aq-2_	R^2^	IC_50 eth-2_	R^2^	IC_50 aq-3_	R^2^	IC_50 eth-3_	R^2^
SF1	0.483*	0.988	0.27	0.999	0.5647	0.979	0.1	0.973	0.534	0.991	0.15	0.994
SF2	0.645	0.981	0.28	0.947	0.556	0.991	0.181	0.901	0.85	0.902	0.16	0.937
SF3	0.648	0.997	0.19	0.94	0.528*	0.987	0.18	0.944	0.775	0.961	0.14	0.995
SF4	0.868	0.982	0.08*	0.98	1.039	0.876	0.09*	0.987	0.579	0.968	0.15	0.968
SF5	0.799	0.971	0.15	0.911	0.604	0.914	0.12	0.915	0.491*	0.952	0.12*	0.981
BHT	0.42	0.9248										
Rutin	0.26	0.9404										

Note: aq-1 = aqueous pre-flowering, aq-2 = aqueous flowering, aq-3 = aqueous post-flowering; eth-1 = ethanol pre-flowering; eth-2 = ethanol flowering; eth-3 = ethanol post-flowering.

* indicates extract with lowest IC_50_ value.

#### Percentage DPPH scavenging activity

**Figure 5:**
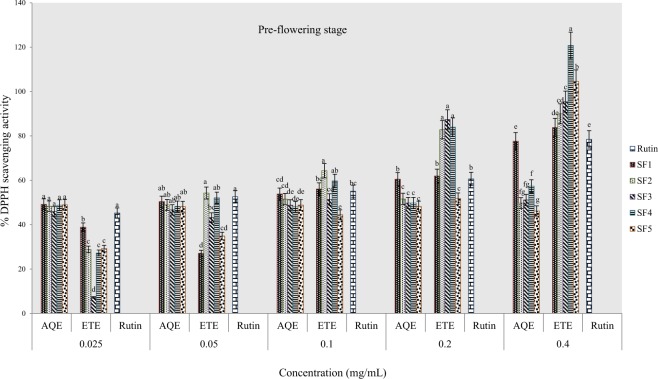
Effects of soil types on % DPPH scavenging activity in *A. caudatus* at the pre-flowering stage.

**Figure 6:**
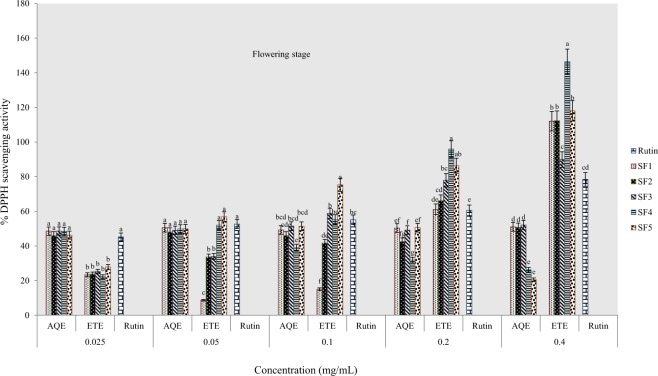
Effects of soil types on % DPPH scavenging activity in *A. caudatus* at flowering stage.

**Figure 7:**
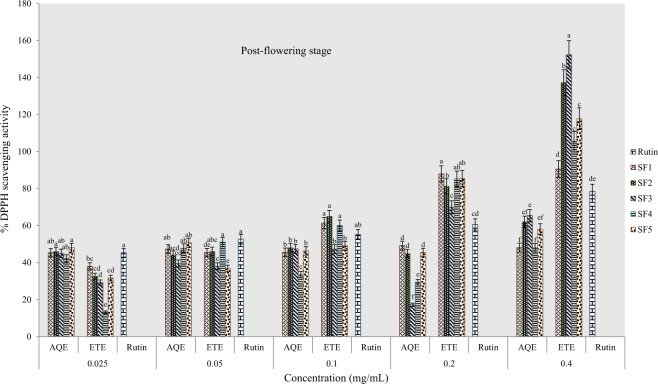
Effects of soil types on % DPPH scavenging activity in *A. caudatus* at the post-flowering stage.

The DPPH radical scavenging activity of tested samples revealed variations in scavenging capacity of extracts from various formulated soils and solvents of extraction. The standard antioxidant drug used was rutin with concentrations ranging from 0.005–0.08 mg/mL. Results obtained indicate that the scavenging activity was neither concentration nor solvent dependent. At the pre-flowering stage, the percentage scavenging capacity was highest and lowest respectively in ethanol extracts of clayey loam and control soils (Fig. 5). In addition, all ethanol extracts were more active than the rutin standard (at highest concentration) although the highest percentage scavenging was recorded in aqueous extracts of the control soil. Similarly, ethanolic extract from clayey loam soil exhibited the highest scavenging activity at flowering stage whereas; the standard was more active than the aqueous extracts (Fig. 6). At the post-flowering stage, ethanol and aqueous extracts of sandy clayey loam were more active than samples from other soil types, however, the rutin standard was more active than the aqueous samples (Fig. 7).

Results of graded series of sample concentrations were used to estimate the concentrations at which fifty per cent of DPPH radicals (IC_50_) had been scavenged. In the ethanolic extract of pre-flowering stage harvest, IC_50_ values ranged from 0.009 mg/mL in sandy clayey loam to 0.075 mg/mL in clayey loam and in water extract, values ranged from 0.0078 mg/mL in control soil to the highest value found in loam (Table 3). At flowering stage, IC_50_ values ranged between 0.01 mg/mL in clayey loam and 0.076 mg/mL in sandy clayey loam of water samples and in ethanolic samples, value ranged from 0.008 mg/mL in loam to 0.035 mg/mL in control soil. The lowest IC_50_ value of 0.009 mg/mL was recorded in water sample of loam in the post-flowering harvest as against the highest values in SF_4_ and SF_1_ wherein both cases; IC_50_ was greater than 0.08 mg/mL. Also in ethanolic extracts, IC_50_ ranged from 0.0097 to 0.022 mg/mL at post-flowering stage. This assay also indicates that the most active extract was from clayey loam (SF_4_) at the pre-flowering and flowering stages. It further confirmed that the extract was more active than rutin.

**Table 3:** IC_50_ data for DPPH scavenging activity of *A. caudatus* cultivated on formulated soils.

Soil types	Pre-flowering				Flowering				Post-flowering			
	IC_50 aq-1_	R^2^	IC_50 eth-1_	R^2^	IC_50 aq-2_	R^2^	IC_50 eth-2_	R^2^	IC_50 aq-3_	R^2^	IC_50 eth-3_	R^2^
SF1	0.0078*	0.509	0.018	0.858	0.014	0.533	0.035	0.935	>0.08	0.509	0.013	0.946
SF2	0.014	0.932	0.009	0.964	0.076	0.781	0.026	0.998	0.052	0.766	0.012	0.986
SF3	0.047	0.735	0.018	0.954	0.014	0.502	0.017	0.98	0.067	0.735	0.022	0.978
SF4	0.041	0.811	0.0048*	0.787	0.01*	0.926	0.009	0.965	>0.08	0.811	0.0097*	0.97
SF5	>0.08	0.917	0.035	0.965	0.011	0.998	0.008*	0.976	0.009*	0.917	0.02	0.973
Rutin	0.008	0.9716										

^*^ indicates extract with a lowest IC_50_ value.

#### Total antioxidant capacity (phosphomolybdate assay)

**Figure 8:**
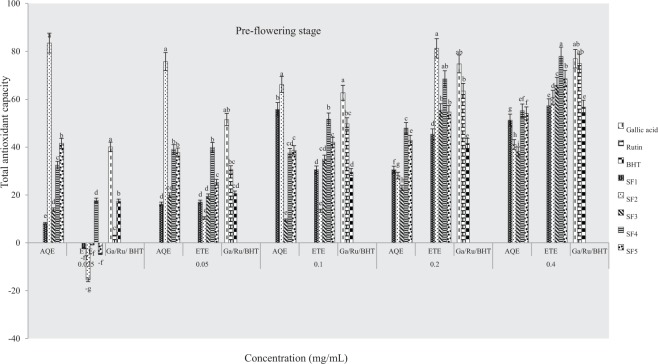
Effects of soil types on total antioxidant capacity in *A. caudatus* at the pre-flowering stage.

**Figure 9:**
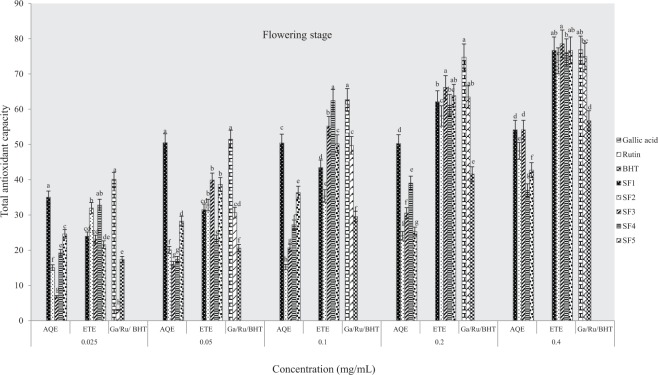
Effects of soil types on total antioxidant capacity in *A. caudatus* at flowering stage.

**Figure 10:**
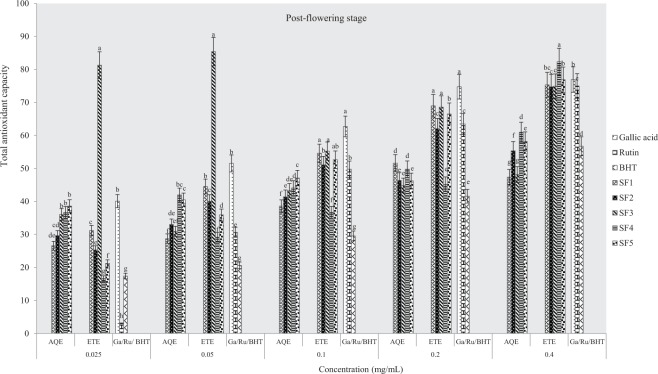
Effects of soil types on total antioxidant capacity in *A. caudatus* at the post-flowering stage.

The total antioxidant capacities of plant samples and standard drugs (gallic acid, rutin and BHT) were also evaluated using phosphomolybdate assay. Figures (8–10) indicate antioxidant activities of ethanol and aqueous extracts at pre-flowering, flowering and post-flowering stages. Pre-flowering samples show that the total antioxidant activity was neither solvent nor concentration-dependent. At the flowering stage, ethanolic extracts had the highest activity in sandy clayey loam and were more active than water extracts and standards. At the highest concentration, ethanol extract of clayey loam was more active than other post-flowering samples evaluated (8–10). Furthermore, the lowest IC_50_ was observed in an aqueous extract from control soil and an ethanolic sample of clayey loam at pre-flowering stage (Table 4). At flowering and flowering stages, the IC_50_ were lowest in aqueous extracts of control soil and ethanolic samples of sandy clayey loam. This assay, therefore, indicates that the most active extract was from clayey loam (SF_4_) at pre-flowering and flowering stages. It further confirmed that antioxidant activities of plant extracts were comparable to rutin and gallic acid but higher than BHT.

**Table 4:** IC_50_ values for total antioxidant capacity of *A. caudatus* cultivated on formulated soils.

Soil types	Pre-flowering				Flowering				Post-flowering			
	IC50 _aq-1_	R^2^	IC50 _eth-1_	R^2^	IC50 _aq-2_	R^2^	IC50 _eth-2_	R^2^	IC50 _aq-3_	R^2^	IC50 _eth-3_	R^2^
SF1	0.093*	0.576	0.278	0.993	0.048*	0.647	0.134	0.979	0.188*	0.989	0.077	0.987
SF2	0.142	0.945	0.263	0.961	>0.4	0.91	0.163	0.953	0.279	0.997	0.094	0.981
SF3	>0.4	0.846	0.175	0.991	0.363	0.983	0.083*	0.993	>0.4	0.43	<0.025*	0.74
SF4	0.256	0.92	0.093*	0.982	>0.4	0.946	0.085	0.788	0.202	0.973	0.226	0.976
SF5	0.328	0.95	0.16	0.964	>0.4	0.518	0.101	0.996	0.261	0.994	0.092	0.918
Gallic acid	0.046	0.9652										
Rutin	0.101	0.9674										
BHT	0.309	0.9715										

^*^ indicates extract with a lowest IC_50_ value.

#### ABTS scavenging capacity

**Figure 11:**
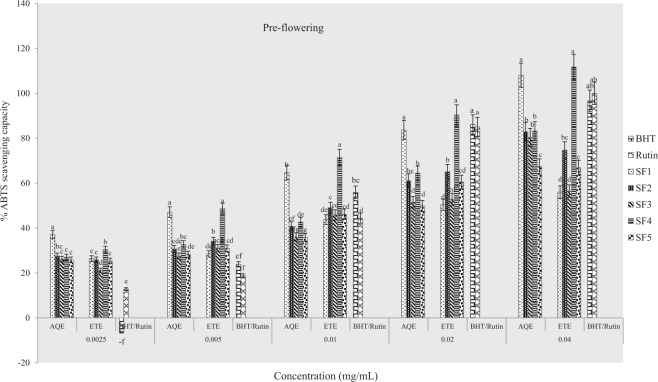
Effects of soil types on % ABTS scavenging capacity of *A. caudatus* at the pre-flowering stage.

**Figure 12:**
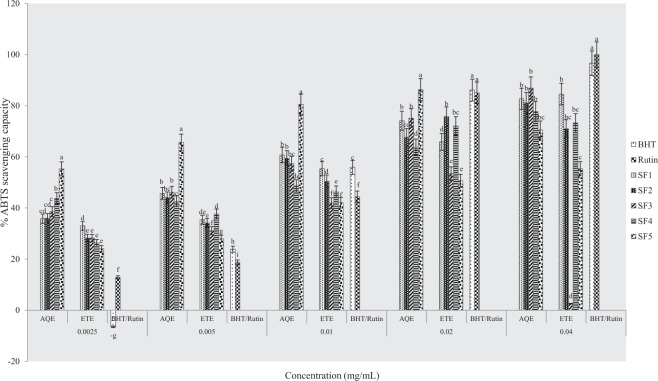
Effects of soil types on % ABTS scavenging capacity of *A. caudatus* at flowering stage.

**Figure 13:**
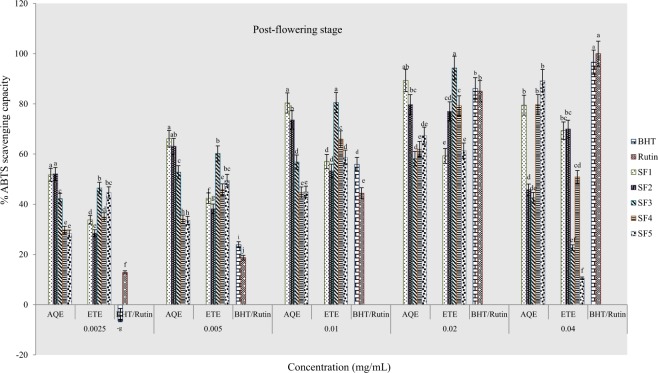
Effects of soil types on % ABTS scavenging capacity of *A. caudatus* at the post-flowering stage.

Results of percentage ABTS scavenging activities of extracts and standard drugs are presented in Figs (11–13). The highest percentages of scavenging activities were observed in ethanolic and aqueous extracts from clayey loam and control soils respectively at the pre-flowering stage. At the flowering stage, aqueous samples of sandy clayey loam showed the highest scavenging activity and at post-flowering, ethanolic extract of sandy clay loam had the highest activity. Table 5 below presents IC_50_ data of ABTS scavenging activities of plant samples. Lowest IC_50_ values were observed in ethanolic extract of clayey loam at the pre-flowering stage, aqueous extracts of loam at flowering stage and an aqueous sample of control soil at post-flowering. This assay, therefore, indicates that the most active extract was from clayey loam (SF_4_) at the pre-flowering stage. It further confirmed that antioxidant activities of plant extracts were comparable to rutin and BHT at the pre-flowering stage.

**Table 5:** IC_50_ values for percentage ABTS scavenging activity of *A. caudatus* cultivated on formulated soils.

Soil types	Pre-flowering				Flowering				Post-flowering			
	IC_50 aq-1_	R^2^	IC_50 eth-1_	R^2^	IC_50 aq-2_	R^2^	IC_50 eth-2_	R^2^	IC_50 aq-3_	R^2^	IC_50 eth-3_	R^2^
SF1	0.0057*	0.9457	0.0196	0.9526	0.0063	0.9924	0.008*	0.9594	<0.0025*	0.9505	0.0076*	0.9653
SF2	0.0144	0.9786	0.0105	0.9892	0.0069	0.9912	0.0099	0.992	0.037	0.9863	0.0089	0.9982
SF3	0.0189	0.9985	0.0157	0.9542	0.0067	0.9821	0.017	0.9849	0.0067	0.8773	0.017	0.9998
SF4	0.0133	0.9605	0.0049*	0.9991	0.011	0.9692	0.011	0.9408	0.0105	0.9648	0.0112	0.9911
SF5	0.02	0.9863	0.0127	0.9761	<0.0025*	0.9508	0.019	0.9663	0.122	0.9686	0.019	0.9995
Rutin	0.011	0.9502										
BHT	0.015	0.9443										

^*^ indicates extract with a lowest IC_50_ value.

## Discussion

Therapeutic activity of plants is a reflection of their phytochemical richness^27^. The type of phytochemicals present in a plant affects its antioxidant activity. These phytochemicals include flavonoids, phenolic acids and alkaloids among others which play overlapping roles in plant defence mechanisms, pollinators attraction, singlet oxygen scavengers, high energy radiation absorbers, reducing agents, allelopathy and sometimes as transition metal chelators^28–32^.

The effect of soil on quantity and relative activity of these phytochemicals cannot be underestimated. A combination of mineral resources provided externally by the soil and internal nutrient trade-offs dictate plant’s carbon-nutrient balance that influences synthesis and retention of defensive chemicals in plants^33^. This justified the need to assess phytochemical variations in *Amaranthus caudatus* cultivated on different soils to document the soil with the highest phytochemical yield considering various reports that stages of growth affect phytochemical and antioxidant properties of plants^34,35^.

The following factors namely; soil texture, bulk density and soil carbon (organic matter) vary considerably in undisturbed soil and they influence soil porosity^7^. Soils used for planting in this experiment had a varying proportion of sand, silt and clay in their formulation. Low bulk density and high organic matter have been attributed to high clay content as well as high phenolic acids^36^. This further justifies the overall high phenolic content obtained in a plant grown on clayey loam soil. Also, the clayey loam soil has the highest water retention property than other soils investigated due to its highest clay content (Table 1). This may have restricted the carbon/nutrient trade-offs due to the dilution effect, hence, concentrating secondary metabolites in the plant.

Comparing the potency of extracts derived from the two extractants used, ethanolic extracts showed higher activity than water. This may be due to varying degrees of polarity and eluent strength of water and ethanol. Ethanol has been reported to be appropriate for extraction of compounds with wide range polarity while water is suitable for compounds with strong polarity^37^. Thus, a higher quantity of phenolic compounds, proanthocyanidin and flavonoids were observed in ethanol samples than water hence, better activity. This agrees with other reports that ethanol is more suitable for the extraction of phenolic compounds in plants^37,38^.

Also, total flavonoid and phenolic compositions of the investigated species varied with growth stages and soil types. Riipi *et al*. (2002) hypothesised that at flowering, differentiation dominates over the synthesis of secondary metabolites. Hence, the highest phytochemical yield recorded in samples harvested from clayey loam at the pre-flowering stage is in agreement with this hypothesis^33^. This agrees further with prior reports that protein-bound phenols, tannic polyphenols, and digestibility of organic matter decrease as plants approach reproductive stages^39,40^. Comparing this study to previous reports on *A. caudatus*, the total phenolic contents recorded were higher than previous data. For instance, Li *et al*.^15^, Pieretti *et al*.^18^, Jiménez-Aguilar and Grusak^16^ and Enujiugha *et al*.^41^ respectively reported 14.94 ± 0.32, 4.35 ± 0.19, 4.1 ± 1.1 and 0.007 ± 0.003 mg (GAE/g) of phenolic acids in different samples of *A. caudatus* as against the highest (104.87 ± 5.93 mg GAE/g) and the lowest (17.94 ± 1.04 mg GAE/g)) phenolic content recorded in this experiment. In contrast, Azeez *et al*.^42^ reported a high phenolic acid of 101.63 ± 0.58 mg GAE/g in his *A. caudatus* sample which matches the phenolic acids content evaluated in pre-flowering samples from all soil types. Similarly, the flavonoid (54.75 ± 0.35 mg QE/g) reported by Azeez *et al*.^42^ compares with pre-flowering flavonoids recorded in this study, probably due to the fact that the plant was harvested after four weeks (before flowering). Moreover, the proanthocyanidins in samples investigated in this study were higher than the earlier record for the same plant. Previously, Jo *et al*.^43^ and Enujiugha *et al*.^41^ respectively had reported lower values of proanthocyanidin (0.516 ± 0.02 and 0.045 ± 0.000 mg TAE/g) in *A. caudatus* using the tannic acid standard as opposed catechin standard used in this study.

Furthermore, the results of antioxidant activities of the plant extracts evaluated through DPPH, NO, ABTS and phosphomolybdate assays elucidate the direct relationship between phytochemicals and antioxidant activities. This is in agreement with postulations in^44–47^ that phytochemicals are precursors to biological activities in plants. Moreover, similar trends observed in the percentage scavenging and IC_50_ values of pre-flowering extracts when tested with DPPH^+^ and ABTS^+^ radicals may have to do with a high degree of solubility of the radicals in water and ethanol. Hence, the same mechanism of action of the two radicals in transferring labile hydrogen atoms^48^. The low IC_50_ values of DPPH^+^ and ABTS^+^ radicals in this study (Tables 3, 5) confirms that samples possess potent antioxidant effect, giving rise to the extremely high percentage of scavenging (Figs 5–7, 11–13) recorded when extracts were tested with the two radicals. Lower percentages of scavenging in DPPH^+^ (77.5% and 66.3%) and ABTS^+^ (48.75 ± 1.1 and 87.1%) were respectively reported earlier by Ashok Kumar *et al*.^19^ and Jo *et al*.^43^.

Nitric oxide (NO) is a reactive oxygen species that has been thought to resemble a mediator of responses produced by vascular endothelial cells. When reacted with superoxide (O_2_^−^) in the epithelium, it may lead to blood pressure and oxidative damage of DNA due to the formation of ONOO^−^ that has the tendency to mutilate supercoiled DNA structure^13,49^. Extracts of *A. caudatus* showed a high propensity to inhibit NO especially in the ethanolic samples where at times, activity was about three times higher than aqueous samples. For phosphomolybdate assay, percentage scavenging activity was neither solvent nor concentration-dependent. The same lowest IC_50_ value of 0.093 was obtained in an aqueous sample of control soil and ethanolic extract of clayey loam at pre-flowering stage (Table 4). In addition, results for inhibition of NO in this study present a higher inhibitory effect compared with what was reported in^19^ for the same plant. Therefore, variations obtained in antioxidant activities of crude extracts of harvests from different soils support some reports that soil environment affects the concentration of phytochemicals and antioxidant gradient^47,50,51^.

## Conclusion

Results from this study suggest that soil types and harvest stage affect phytochemicals and antioxidant properties of *Amaranthus caudatus*. The biological activity of the plant extract also depends on the solvent of extraction, stage of maturity, soil type and partly, the choice of the assay. All phytochemicals assessed were highest in the clayey loam at the pre-flowering stage. The same trend was replicated in the antioxidant activities evaluated, thus; flavonoids and phenolic acid contents contribute significantly to bioactive makeup of a plant. Based on this, it is recommended that for optimal biological potency, *A. caudatus* should be cultivated in clayey loam soil and harvested prior to flowering. Also, for pharmacological purposes, ethanol is preferred as a solvent of extraction. Thus, the vegetable should be consumed regularly for health improvement since it exhibited higher antioxidant activities more than known antioxidant drugs such as BHT, rutin and gallic acid.

## Data Availability

The datasets generated and/or analysed during the current study are available from the corresponding author on reasonable request.
